# Outsourcing Transcription: Extending Ethical Considerations in Qualitative Research

**DOI:** 10.1177/10497323221101709

**Published:** 2022-05-21

**Authors:** Marita Hennessy, Rebecca Dennehy, Jennifer Doherty, Keelin O’Donoghue

**Affiliations:** 1 Pregnancy Loss Research Group, Department of Obstetrics and Gynaecology, 8795 University College Cork, Cork, Ireland; 2 INFANT Research Centre, University College Cork, Cork, Ireland; 3 College of Medicine and Health, 8795 University College Cork, Cork, Ireland; 4 JD Audio Transcription, Donegal, Ireland

**Keywords:** ethics, qualitative, research ethics, transcription, vicarious trauma

## Abstract

Research ethics considerations foreground minimising harm to participants. Whilst increasing attention is being paid to researcher vulnerabilities, little has been written about transcriptionists, who can potentially experience emotional distress and vicarious trauma. In this article, we highlight ethical considerations when outsourcing audio for transcription as part of the RE:CURRENT (REcurrent miscarriage: evaluating CURRENT services) Project. Through qualitative interviews, we explored the perspectives of those involved in the management/delivery of services, and women and men who experienced recurrent miscarriage (*N* = 62). We put distress protocols in place for participants, researchers and the transcriptionist, and adopted a research team approach with the professional transcriber. The transcriptionist highlighted the isolated nature of the role; how researchers often did not brief her when commissioning work, and how the personal impacts of this work were rarely considered. Researchers and ethics committees should consider ethical responsibilities to ‘do no harm’ when it comes to transcriptionist wellbeing.

## Overview

In this article, we highlight and explore ethical issues considered when outsourcing audio for transcription. Firstly, we provide insights into transcribing qualitative data. We then discuss the importance of considering the psychological/emotional needs of transcriptionists, and draw on our experiences – as researchers and a professional transcriptionist – transcribing audio for a qualitative study of stakeholders’ views of services and supports for recurrent miscarriage in the Republic of Ireland. Finally, we discuss key learnings and provide suggestions for others who may be considering outsourcing transcription and/or who may not have considered the impact of the endeavour on those outside of the research team.

## Transcribing Qualitative Data

Transcription is an interpretive act (Clark et al., 2017; Green et al., 1997; Lapadat & Lindsay, 1999; Tilley & Powick, 2002), and also a situated act as researchers locate themselves within the context of their own assumptions about language and culture and discourse practices (Bird, 2005; Green et al., 1997; Tilley, 2003a). Researchers have described their own experiences and insights into transcribing qualitative data (Bird, 2005; Oliver et al., 2005), seeing it as ‘both as product and as methodological process’ (Bird, 2005). Some researchers have interviewed transcribers about their experiences of the transcription process (Tilley & Powick, 2002; Wilkes et al., 2015), others have published on both (Tilley, 2003a, 2003b) and also reported the value of insights from transcribers into the analysis/interpretation of data (Etherington, 2007; Tilley, 2003a).

Audio transcription is poorly addressed in general, if at all, in qualitative textbooks, journal articles and elsewhere (Braun & Clarke, 2013a; Davidson, 2009). Many researchers highlight the value of doing your own transcribing, to build intimate knowledge of your data and starting the analytical process (Bazeley, 2013; Braun & Clarke, 2013a; Richards, 2015). Researchers are encouraged to consider such merits against any constraints such as their time and resources (Richards, 2015). Much literature focuses on how to approach doing transcription (Davidson, 2009). When outsourcing transcription, Bazeley (2013) suggests providing clear, written decision and formatting guidelines, potentially asking the transcriber to note their reflections on the content of the audio as they transcribe, and ensuring the transcriptionist follows other instructions, including those concerning confidentiality. She also recommends reviewing and editing transcripts whilst listening to the recordings as typist interpretations can change the intended meaning of the written words. Braun & Clarke, 2013a provide detailed guidance on data transcription, but from the perspective that the researcher is undertaking it themselves; they do however provide sample transcriber confidentiality agreements in the companion website (Braun & Clarke, 2013b). Guidance on establishing interview transcription and translation protocols for research on sensitive topics has also been published, focussing on similar topics as those outlined above, as well as managing and translating transcripts (Clark et al., 2017). There is limited guidance within the literature in general however around ethical issues outsourcing transcription, particularly transcriptionist support needs.

## Why it is important to consider the psychological/emotional needs of transcriptionists?

Most research ethics discussions centre around minimising harm by placing participant interests at the forefront (Braun & Clarke, 2013a). A broader consideration of ethical implications is required to be an ethical researcher however, rather than simply attending to minimum standards within ethical codes of conduct (Braun & Clarke, 2013a, p. 61). The potential vulnerabilities of researchers also need to be considered; we often gather in-depth, detailed data about sensitive issues, can be traumatised by the data we collect/generate, and can undertake data collection within contexts where there can be concerns for our physical safety. Risk of physical and psychological harm to researchers are receiving more attention (Bashir, 2020; Keyel, 2020; Rager, 2005). For example Bashir (2020) describes how researchers can feel exposed in situations beyond their control and unprepared to deal with the settings, information, circumstances and/or emotions in such encounters, and beyond. Twenty years ago, guidelines for the safety of social researchers were issued by the Social Research Association (2001). Little attention, however, has been paid to the psychological safety of transcriptionists (Kiyimba & O’Reilly, 2016a, 2016b), who can potentially experience secondary stress and vicarious trauma (Kiyimba & O’Reilly, 2016b) which may be similar to the experiences of professional interpreters (Geiling et al., 2021; Lai & Costello, 2020). Transcriptionists may also work on different projects, successively or in parallel, which may be of a similar nature, with the potential for long-term impacts. The lack of attention to transcriptionists is perhaps unsurprising, given the lack of status afforded to transcription work (MacLean et al., 2004; Tilley & Powick, 2002).

There is a need for researchers and others to consider ethical responsibilities to ‘do no harm’ when it comes to professional transcribers (Etherington, 2007; Wilkes et al., 2015). Whilst this ethical principle was developed in the context of medical practice and since extended to biomedical and social science research in terms of protecting patients and /or research participants (Beauchamp & Childress, 2019; Slowther et al., 2006; World Medical Association, 2001), we argue that this principle should be extended to professional transcribers. It is a core ethical principle for healthcare research and, as highlighted by Slowther et al. (2006, p. 66), forms part of ‘the duty of care that a researcher owes to research participants, and the duty that a research institution or sponsor owes to both participants and researchers’. In the latter instance, we broaden the interpretation of researcher to include those contracted by the researcher, research team and/or sponsor to conduct part of the research, including transcriptionists.

Transcriptionists can become emotionally connected to the ‘characters they construct based on the tapes’ (Tilley, 2003a, p. 764). They can also experience emotional distress and feelings of helplessness (Etherington, 2007; Kiyimba & O’Reilly, 2016a; Wilkes et al., 2015). Emotional consequences for transcriptionists include both ‘emotional labour’ (dealing with the face-to-face/voice-to-voice contact and concern for those whose voices they were hearing) and the ‘emotional work’ (dealing with the emotions of others /empathy and concern for researchers’ physical and emotional wellbeing) (Kiyimba & O’Reilly, 2016a). Thus, transcribing our research interviews can potentially cause harm to transcriptionists and we have a duty to negate or minimise such harms. In their research with professional transcriptionists, Kiyimba and O’Reilly (2016a) also found that a lack of safeguarding protocols makes the role very challenging despite some use of coping skills such as acceptance, compartmentalising, desensitisation, rationalisation and detachment. This was also noted by Wilkes et al. (2015). The authors recommended the introduction of a research team approach as an additional safeguard for transcriptionists; a similar recommendation has been made with regard to professional interpreters (Lai & Costello, 2020). In particular, they felt that they should be briefed about any potentially distressing content to the recordings that they are transcribing, as this would provide an opportunity to make an informed decision regarding taking on the role and prepare them for any potential emotional impact (Kiyimba & O’Reilly, 2016a). MacLean et al. (2004) also highlight the impacts of emotionally laden content on transcriptionists, on a personal level and on the quality of transcripts produced; they advocate that necessary protocols are put in place, including briefing and debriefing. The emotional impact is important to consider as transcriptionists tend to work in a more isolated manner than researchers (Kiyimba & O’Reilly, 2016a).

## Interviews undertaken as part of the RE:CURRENT Project

The RE:CURRENT (REcurrent miscarriage: evaluating CURRENT service) Project is a two-year programme of work (2020–2022) being undertaken to evaluate recurrent miscarriage services in the Republic of Ireland. Recurrent miscarriage involves the loss of two (ESHRE Early Pregnancy Guideline Development Group, 2017), or three (Royal College of Obstetricians and Gynaecologists, 2011), consecutive pregnancies or more, and affects 1–3% of the reproductive age population (Quenby et al., 2021). There are many uncertainties around investigations and treatments (Coomarasamy et al., 2021), and recurrent miscarriage is a significant source of psychological distress for women/couples (Quenby et al., 2021). Whilst some evidence-based treatments have improved the outcomes for couples, almost half of cases remain unexplained (Clifford et al., 1994). Currently there is no national standard for the management, investigation or follow-up of those who experience recurrent miscarriage in Ireland. Findings from RE:CURRENT will inform efforts to standardise and improve the quality of recurrent miscarriage services and supports.

One of the RE:CURRENT work packages involves examining multiple stakeholder views on the provision of recurrent miscarriage services and supports (including investigations, treatment and follow-up), as well as the experiences of those who engage with such services. From June 2020 to January 2021, we interviewed 42 individuals involved in the delivery and management/governance of services and supports (including consultant obstetricians and gynaecologists, specialist registrars, clinical midwife/nurse bereavement specialists, midwives, sonographers, medical social workers, public health nurses and general practitioners, as well as representatives from advocacy and support organisations, and those involved in the administration, governance and management of maternity services), and 20 women and men who had experience of at least two consecutive miscarriages in the 24 months prior to participation (Dennehy et al., 2022).

In our ethics application, we noted details of ethical considerations regarding participants in line with the Declaration of Helsinki (World Medical Association, 2001) and the four key principles of biomedical ethics, namely respect for autonomy, nonmaleficence, beneficence and justice (Beauchamp & Childress, 2019). This included attending to informed consent, confidentiality, security and privacy during virtual interviews, data handling and storage, any questions/concerns the participants may have, and having a distress protocol in place. We advised participants that they could pause or cease the interview at any time; the research team also used their judgement to determine if it was necessary to pause or cease an interview (Orb et al., 2001). We conducted debriefing with participants at the end of each interview to ensure their wellbeing, particularly given the nature of the discussions which were potentially distressing. If required, we outlined/provided supports according to our participant distress protocol. Within our protocol, participants are initially encouraged to contact a family member or friend for support before the end of the session. With their permission, the researcher checks-in with them within 24 hours, and provides details of support networks, and, where deemed necessary, they are referred to the clinical nurse/midwife bereavement specialist, or medical social worker, in the relevant maternity unit/hospital.

We also had a distress protocol to ensure the safety of members of the research team, all experienced in qualitative data collection with ‘vulnerable' populations and on sensitive topics including pregnancy loss (Dennehy et al., 2019; Meaney et al., 2017). Interviews were conducted by RD and MH, both experienced qualitative interviewers/researchers. Following each interview, RD contacted MH, or vice versa, and they debriefed. If distressed following an interview, they could also debrief with a senior member of the research team. If further support was required, they would be directed to counselling services. Field notes recording contextual information and initial interpretations/reflections were written following each interview (Phillippi & Lauderdale, 2018), and upon listening to interview recordings and further analysis. These informed the ongoing development of the study, researcher reflexivity and analysis.

## Outsourcing transcription in the RE:CURRENT Project

We sought the services of an external professional company specialising in transcription services to transcribe audio files, given the volume of data generated and the tight project timelines. Following the sourcing of quotes in line with our university’s procurement processes we secured the services of JD Audio Transcription in January 2020, and the lead researcher (RD) advised JD that we would be in contact again once data collection was underway. Subsequently, in June 2020, RD contacted JD – by phone, with follow-up email – to outline the project and timelines, check her availability and highlight that if JD experienced any issues, to get in touch. JD also signed a non-disclosure agreement. Transcription processes such as approach/formatting and spot checks were also agreed. As they were generated, RD transferred audio files to JD for verbatim transcription throughout the period of June 2020 to February 2021. There was ongoing dialogue/communication between RD and JD about the nature of the interviews, the transcription process and any issues arising for JD. Interviews with those involved in the delivery and management/governance of services and supports were conducted and transcribed first. Once completed, RD and MH had a virtual meeting with JD to discuss her experience of transcribing the interviews and the next phase of transcribing interviews with women and men with lived experience of recurrent miscarriage; they also held discussions with JD after transcription of the latter data was completed. JD contacted RD unprompted, on one occasion during the latter phase of transcription. See Box 1 for an overview of JD’s perspective on the approach taken in the RE:CURRENT Project and her experiences. This was written by JD in June 2021 following completion of the work and an invitation by MH to write a short reflective piece on her experiences to inform the writing of this paper and ensure that her perspective was incorporated in a meaningful way. MH and JD spoke briefly over the phone to discuss what this would involve (i.e. describing how she experienced the process). A week later JD forwarded her reflection which appears, unedited, in Box 1.

MH had used the services of JD Audio Transcription previously to transcribe audio of interviews with parents of children aged under two years about their views and experiences of promoting ‘healthy’ growth and associated behaviours (Hennessy et al., 2020). She did not consider any potential for distress on the part of the external transcriber when commissioning her services; neither did any issues become apparent as transcription progressed. In the RE:CURRENT Project, however, MH and RD were cognisant of any potential impact on the external transcriber, as all members of the research team were of potential impacts on both participants and interviewers alike.

**Box 1 A transcriptionist’s perspective: JD, owner of JD Audio Transcription, established in 2017**

I was first contacted by Rebecca Dennehy in June 2020, following the commissioning of my services. We spoke on the phone and she outlined the aims and objectives of the RE:CURRENT study and what was required from me. I had a clear outline of the timeline and the topic before I signed the non-disclosure agreement and began transcribing the audio interviews. Often people will contact me by email without giving me any information about their research topic. The only way I can get an insight, if I don’t ask them, is by the title in the signature of their email. So much communication is by email nowadays and working from home as a transcriber can be an isolating experience. If you aren’t careful you could go days without speaking to people. It was lovely to speak regularly to RD and MH throughout the process and even more surprising when they asked for feedback on the difficult subject matter and how it affected me as a transcriber. I had always transcribed audio without considering the personal impact of sensitive material, so I never expected the researchers to consider my feelings either. I am a people person, and sociology or psychology-based topics are what I enjoy transcribing most. I like hearing the human stories although at times I can find it hard to switch off. I don’t just type the words and forget about them. Maybe this is a skill that other transcribers have mastered better than me. Pregnancy and recurrent miscarriage are not topics that I have direct experience of so I probably couldn’t emotionally relate to them in the same way as another topic might. However, some of the interviews with the parents were difficult to listen to particularly as I was hearing them tell their own story in their own words through audio rather than reading the text. The experience of transcribing the research was much less stressful because I knew I was supported if any issues did arise. I believe it is a privilege when someone trusts me to transcribe sensitive data and I always treat it with confidentiality and respect. It is an added bonus when the researcher considers the transcriber’s feelings in this process as well.

## Discussion

In this article, we have drawn on our experiences within a large qualitative research project which examined the perspectives of those involved in the management/delivery of recurrent miscarriage services and supports, and women and men with lived experience, to highlight ethical issues relating to the outsourcing of transcription, particularly on transcriptionist wellbeing.

Transcription is generally a neglected aspect of written reports of research, with authors often just noting that interview data were transcribed (Davidson, 2009). It is difficult to ascertain who conducts transcription – whether it is the researcher themselves, a professional/external transcriptionist (whether a sole trader or employed within a large transcription service), someone (e.g. a student/other researcher) working within the same organisation but separate to the researcher/research team, or whether speech recognition software has been used, which is becoming more common (Duca, 2019) though is not without limitations (MacLean et al., 2004). As noted in other research, the transcriptionist in our study worked in a more isolated manner than researchers (Kiyimba & O’Reilly, 2016a). MacLean et al. (2004) note that most transcriptionists are women, with many taking on this type of work for supplementary income, or may be employed as secretaries, research or administrative assistants, or clerks in a research or academic environments. They further argue that “These *invisible persons*, however, play a key role in much qualitative research, participating consciously or not in a transformatory auditory experience” and should be recognised as professional members of the research team, adhering to confidentiality and reimbursed appropriately (MacLean et al., 2004, p. 118). Whatever the employment circumstances of the transcriptionist, it is important for researchers to consider impacts on those involved in transcribing data and their duty of care in this regard.

Transcriptionists should be briefed about the project at the outset, including, but not limited to, any potentially distressing content in the audio recordings that they are transcribing. Similar to the process for research participants, this would enable them to make an informed decision about taking on the work (Kiyimba & O’Reilly, 2016a). Appropriate distress protocols, including briefing and debriefing, should be put in place (MacLean et al., 2004) if there are no such procedures within the transcriptionist’s own organisation, as applicable. Researchers should establish what is in place as part of any tendering/pre-contractual (or more informal) processes. In addition to briefing, as outlined above, distress protocols should include debriefing and how this will be structured; for example after a certain number of transcripts and/or as the need arises for the transcriptionist, including if they are distressed during/after transcribing a particular interview. As evidenced in our study, there was also much value in informal check-ins during the transfer of audio files and completed transcripts between the researchers and the transcriptionist. The distress protocol could also detail with whom debriefing will be undertaken; for example with a member of the research team (if an independent researcher) or a colleague (if part of a bigger transcription company/network), and if others will be involved as part of escalation of protocols, for example a more senior member of the research team, or counselling services. Potential risks, and protocols to minimise any harm (as outlined), should be documented within ethics applications, as a requirement.

Such ethical considerations should be given due attention by researchers and ethics committees in all projects which require the outsourcing of audio transcription, not just those projects/topics deemed relevant by the researcher/research team. It was interesting to note that MH did not consider any potential for distress on the part of the external transcriber when commissioning her services previously (for pregnancy/early parenthood research), but did for the current project which focused on pregnancy loss. Jennifer Doherty noted that she not have direct experience of the topic, which may have impacted on her (non)emotional engagement with it, unlike experiences documented by Tilley (2003a). All members of the research team, including transcriptionists, should reflect on their positionality at the outset of a project, and throughout, as positionality can change (Hennessy et al., 2022). This will also contribute to informed decision-making, and how distress protocols are enacted. There may be challenges in implementing disclosure procedures which should be acknowledged; transcriptionists – regardless of their employment circumstances – may be reluctant to share any negative experiences and/or impacts for fear of jeopardising their ongoing and/or future employment. Researchers should reiterate the importance of sharing such information and/or debriefing, and include transcriptionists as members of the research team. Furthermore, by increasing awareness of the potential for transcriptionists to experience secondary stress and vicarious trauma and the need to implement appropriate protocols, as we aim to do here, we may contribute to fostering more open and supportive workplaces for transcriptionists in general. This could be reinforced through the development of codes of practice and attention to ‘transcription ethics’ within undergraduate and postgraduate training programmes.

Whilst this article draws on a single example of outsourcing transcription, we nonetheless believe that it highlights key issues pertaining to ethics and protecting the wellbeing of professional/external transcriptionists, given the approach taken, topic under study and the large volume of data transcribed. We share calls from other researchers for research ethics committees to ensure that the psychological wellbeing of transcriptionists, as well as researchers, is accounted for (Kiyimba & O’Reilly, 2016a; Wilkes et al., 2015). We hope this article will encourage consideration and discussion around ethics in outsourcing transcription, amongst researchers and ethics committees alike, and contribute to advancing standards in qualitative research and research ethics.
